# Research Blogs and the Discussion of Scholarly Information

**DOI:** 10.1371/journal.pone.0035869

**Published:** 2012-05-11

**Authors:** Hadas Shema, Judit Bar-Ilan, Mike Thelwall

**Affiliations:** 1 Department of Information Science, Bar-Ilan University, Ramat-Gan, Israel; 2 Statistical Cybermetrics Research Group, School of Technology, University of Wolverhampton, Wolverhampton, United Kingdom; The Centre for Research and Technology, Hellas, Greece

## Abstract

The research blog has become a popular mechanism for the quick discussion of scholarly information. However, unlike peer-reviewed journals, the characteristics of this form of scientific discourse are not well understood, for example in terms of the spread of blogger levels of education, gender and institutional affiliations. In this paper we fill this gap by analyzing a sample of blog posts discussing science via an aggregator called ResearchBlogging.org (RB). ResearchBlogging.org aggregates posts based on peer-reviewed research and allows bloggers to cite their sources in a scholarly manner. We studied the bloggers, blog posts and referenced journals of bloggers who posted at least 20 items. We found that RB bloggers show a preference for papers from high-impact journals and blog mostly about research in the life and behavioral sciences. The most frequently referenced journal sources in the sample were: Science, Nature, PNAS and PLoS One. Most of the bloggers in our sample had active Twitter accounts connected with their blogs, and at least 90% of these accounts connect to at least one other RB-related Twitter account. The average RB blogger in our sample is male, either a graduate student or has been awarded a PhD and blogs under his own name.

## Introduction

The Web has given rise to new forms of scientific discourse. Web 2.0 tools provide scientists with faster, less formal ways for conversation inside and outside the scientific community. Unfortunately, most scientific output created on the Web goes unnoticed by current academic metrics, which measure scientific work published in “conventional” academic literature [1].

Traditionally, evaluation of scholarly work has been often done by citation analysis. Citation indexes work under the assumption that a citation indicates a connection between document A and B, though it does not indicate the nature of the connection [2]. The *normative theory of citations,* suggested by Merton [3], claims that citations are the scientist’s way of acknowledging an intellectual debt to other scholarly works. The *social constructivist* view on citing behavior argues that works are cited for a variety of factors, some of them have nothing to do with intellectual debt (See [4], [5], [6] for a detailed review). For example, open access papers may receive more citations than those behind a paywall [7]. Mentions of academic papers in Web pages are considered Web citations [8], [9]. However, academic Web citations are not necessarily part of the scientific discourse, since they can also be used for other purposes (e.g., navigation aids, self-publicity) [10].

One of the many ways of spreading scholarly information throughout the Web is the research, or science blog. Unfortunately, there has been little research about the way blogs are used by scientists. Most papers dealing with science blogging so far are either opinion pieces [11], [12], interviews with a number of selected bloggers [13], [14]), descriptions of personal experiences as a blogger [15] or content analyses of a relatively small blog sample [16], [17]. There seem to be many different motives behind science blogging: to share content and express opinions, to improve writing skills, to organize thoughts and ideas and to interact and create relationships inside and outside of the author’s home discipline. Science blogging can give the blogger room for creativity and the feeling of being connected to a larger community. It is a means of establishing an online reputation [14]. These motives have much in common with those of medical bloggers: in a survey study [18] 74% of medical bloggers listed “To share practical knowledge and skills” as a motive for blogging and 53% listed the expression of creativity.

Science blogs can add to the transparency of the scientific process by reviewing and discussing the science culture in general and scientific research in particular. They allow informal post-publication peer-review, as well as reviews from people who usually would not be considered “peers”. Organized by two medical writers, Retraction Watch is a blog which covers in detail why peer-reviewed papers are retracted from journals [19]. While journals and authors release announcements regarding retracted papers (which can be as short as “This article has been withdrawn by the authors”) the blog illustrates and adds insights to retractions beyond those found in formal discourse.

Science blogs may influence mainstream science: On December 2, 2010, *Science* published an online paper [20] of NASA scientists claiming to have discovered arsenic-based bacteria. Science bloggers were deeply skeptical about the findings (a collection of blog posts can be found in [21]). Scientists tweeted extensively about the subject under the hashtag #arseniclife. The criticism made its way to articles in mainstream [22]–[24] media outlets, which quoted various blogs. By the time *Science* published technical comments (including one from a blogger [25]) the scientific community online had thoroughly commented and criticized the paper.

Despite the less formal format of blogs, blogging researchers express a desire to refer to papers in their blogs in a scholarly manner [14]. Researchblogging.org (2008), an aggregator of science blogs, allows bloggers to refer to peer-reviewed research in an academic citation format. Bloggers discussing peer-reviewed research can register with the aggregator, and when they mark relevant posts in their blog, these posts appear on the aggregator’s site, allowing one-stop access to research reviews to interested readers. The site’s editors ensure that posts follow the guidelines and are of appropriate quality. Past research found that researchblogging.org (RB) bloggers in the field of chemistry prefer to post about research published in high-impact journals [26]. In the current study, our objective is to learn about RB bloggers in all fields and the type of research they choose to review in order to get insights about scientific blogging in general.

## Methods

Following Groth and Gurney [26] we based our study on data from the science blogs aggregator ResearchBlogging.org. Blogs chosen for the study were non-commercial, written by 1–2 individuals and had a minimum of twenty entries posted at the RB aggregator between January 1, 2010 and January 15, 2011. Twenty posts aggregated in RB ensured that the blogger had a fairly established blog and wrote in an academic manner. A total of 135 bloggers in 126 blogs satisfied our criteria (two bloggers had two blogs each and 11 blogs had two authors each).

We collected the data from the blogs and bloggers’ RB pages as well as the “About” and “Profile” parts of the blogs themselves. If the “About” or “Profile” parts were unclear we searched the Internet for mentions of the blogger’s name in different contexts. The publicly available parts of profiles from LinkedIn, Facebook, Twitter and other social networks, as well as interviews and home pages were used as additional sources of information on the bloggers. All data were manually collected to ensure maximal accuracy. Connections between Twitter accounts were visualized using NodeXL [27], a Microsoft Excel add-on which uses the Twitter API.

We characterized reviewed journal articles in the blog posts based on the bloggers’ last five posts appearing on RB at the time of the data collection in March, 2011. Since almost all of these journals appeared in Thomson-Reuters Journal Citation Reports (JCR), we were able to utilize the JCR categories assigned to these journals. The JCR categories were collated into seven main categories defined by us: life sciences, sciences, medicine, behavioral and neurosciences (incl. psychology and psychiatry), computer science and engineering, social science & humanities and multidisciplinary journals. In a few cases a journal was classified into more than one main category.

The blogs were characterized based on the journals in which the last 10 reviewed papers were published, from July 1, 2011 and backwards. Only papers published in journals indexed by the JCR were taken into account, thus non-indexed articles were skipped and the data collection continued until there was information from 10 items. Papers from multidisciplinary journals were classified according to their title, abstract and key terms used by their journal and/or their repository (e.g. PubMed). One author (JBI) classified papers according to their JCR categories and created the main categories mentioned above. The blog classification was done by another author (HS) with JBI blindly classifying 15% of the blogs as a reliability check. Disagreements were discussed after the primary check until the researchers reached agreement.

## Results and Discussion

### Blog Classification

The blogs were classified in order to map out the most popular blogging fields (Table 1). Life Science blogs were the most popular in our sample, followed by the Psychology, Psychiatry, Neurosciences & Behavioral Science blogs. Blogs about Social Sciences & Humanities and about Computer Science & Engineering were the least represented in our sample.

**Table 1:** The main subject of the 126 Blogs.

Main category*	# category	% category
Life sciences	52	39
Psychology, psychiatry, neurosciences,behavioral sci.	28	21
Medicine	26	19
Sciences	12	9
Multidisciplinary	8	6
Social Sciences & Humanities	7	5
Computer Science & Engineering	2	1

* Nine blogs have two main categories.

RB has its own tagging system, which allows bloggers to classify their posts into one category or more. The biology tag had been found to be the most popular tag in the RB aggregator by a previous study, with 32% of the posts, followed by psychology (13%) and health (12%) [28]. The psychology tag (13%) and the neuroscience tag (8%) amount to 21% of the tags, the same as the Psychology, Psychiatry, Neurosciences & Behavioral Science category in our sample (21%). Our categories and the RB tags are not identical, but overlap enough to give us a crude indication of the resemblance between our sample and the general RB population. In September 2011 the RB aggregator contained around 20,600 posts and about 9,000 of them were tagged “biology”, making it by far the most popular tag.

RB’s tagging system focuses mainly on the life and natural sciences. For example, astronomy has 10 subtags, psychology 21 and biology 28. History, economics and sociology, on the other hand, are represented only as subtags of the “social science” tag. It is possible that the tagging system is a factor in bloggers’ decisions about whether to aggregate in RB, or that the lack of tags shows either a lack of interest of bloggers from those disciplines to aggregate in RB or that they are not familiar with it. Other blogging aggregators (many aggregators are aggregated themselves at http://scienceblogging.org/) might also cater better to those bloggers’ needs. Another possibility is that the RB tagging system merely reflects a reality in which most of the blogging about peer-reviewed research is done in certain fields. The NSF Doctorate Recipients from U.S. Universities report [29] concluded that the number of life science doctorates awarded was rising, which could serve as a partial explanation for the dominance of life sciences blogs and life science papers in our sample. Moreover, according to Bora Zivkovic, Scientific American’s blogs editor “[Blogs are] written by graduate students, postdocs and young faculty, a few by undergraduates and tenured faculty, several by science teachers, and just a few by professional journalists” [30]. Since more than two-thirds of the academic post-doctoral appointments in the U.S. were in the life and medical sciences, it could be that the high number of post-doctorates affects the number of science blogs in those fields [31].

Note that this distribution does not coincide with the distribution of the items published in 2010 and indexed by Elsevier’s Scopus, as can be seen from Table 2. Especially notable are the much higher occurrences of behavioral science and multidisciplinary articles in the blog posts. Due to the limitations of our sample we cannot draw definitive conclusions about whether the general science blogs’ distribution is significantly different from the Scopus items’ distribution.

**Table 2:** Distributions of items published in 2010 and indexed by Scopus.

Main category*	# publications	% of total N = 2,170,251
Life sciences	448,097	21
Psychology, psychiatry, neurosciences,behavioral sci.	95,489	4
Medicine	737,337	34
Sciences	769,316	35
Multidisciplinary	20,397	1
Social Sciences & Humanities	252,759	12
Computer Science & Engineering	653,158	30

* Some items appear in more than one category.

### Gender Distribution

**Figure 1:**
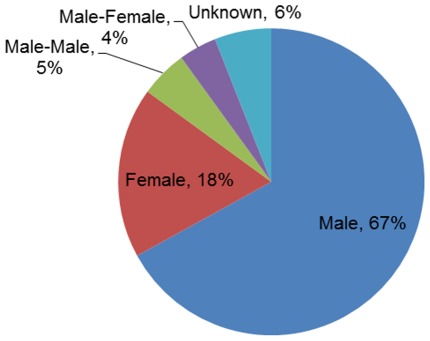
Distribution of gender among bloggers.

In 2009, about 47% of the research doctorates in the U.S. were awarded to women. The percentage of women who were awarded doctorates in the Science & Engineering (S&E) fields went up from 29% in 1989 to 42% in 2009 [31]. Despite the large percentage of doctorates earned by women, men dominate science blogging (Figure 1). About two-thirds of the blogs had one male author, 18% had one female author, 5% had two male authors and 4% had one female and one male author.

The gender disparities in science blogs authorship seem similar to those found in studies of Wikipedia contributors. Glott et al. [32] found that only 12.64% of the contributors to Wikipedia are women. Lam et al. [33] found that the initial percentage of women contributors in their sample was 16.1%, but dropped to around 6% for contributors who have made more than 500 edits. It is possible that our choice of established science blogs has lowered the percentage of women bloggers in our research, since in Wikipedia women’s tenures as editors were shorter than men’s [33]. Our findings are in line with those of Munger [34] who studied the general gender ratio of RB and found *that “male bloggers outnumber female bloggers by over three to one.”*

### Blog Networks

A scientific blog can be an independent venture, or part of a larger group of science blogs. Though these blogs may vary in their subjects and have different authors, they all blog about scientific subjects under one general domain (e.g. http://blogs.plos.org/). Each network has a main portal page featuring various posts from the network’s blogs, as well as links to all the blogs. The British newspaper the Guardian launched its own science blogs network in August 2010 [35] and the PLoS Journals, Wired Magazine and Scientific American subsequently followed suit [36], [37], [38]). The blog networks in our sample, other than Field of Science, were by invitation only. Invitations are usually extended to bloggers already of good standing (the tagline of the Wired science blogs network is “A new network of all-star sciencebloggers.”) [37].

In our sample 87 (69%) were independent blogs and 39 (31%) were part of a bigger group of blogs. Out of the 39 blogs, 15 (38%) belong to one of the three networks run by Seed Magazine (in English, German and Portuguese).

### Twitter

Disseminating scientific knowledge can take place in different Web 2.0 channels. The microblogging service Twitter had 100 million active users by mid-2011 [39], and is being used by some academics for spreading scientific research [40]. Out of the 126 blogs in the sample, ninety (72%) had at least one active, unprotected Twitter account. Blogs which linked to more than one account (in cases of two authors) were counted as one account per blog. The Highly Allochthonous (http://all-geo.org/highlyallochthonous/) blog linked to a combined list of its two authors’ accounts which we counted as a single account. We also found three (2%) protected accounts and six (5%) inactive accounts (tweeted last more than three months before we visited them, on June 2011).

**Figure 2:**
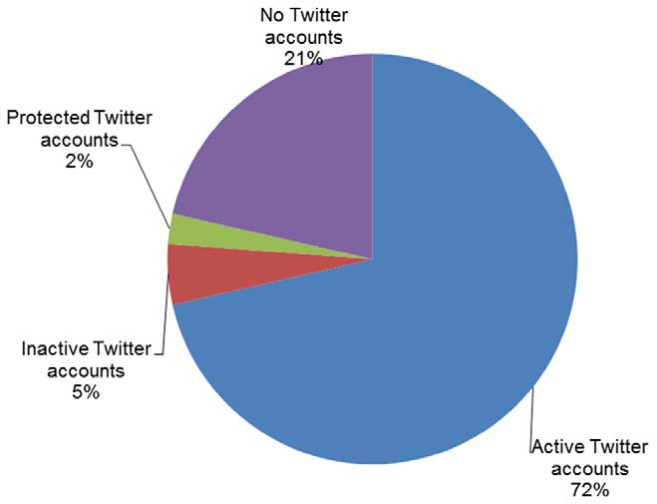
Blogs and Twitter accounts.

Twenty-seven blogs (21%) did not have a Twitter account. The Cognitive Daily blog (http://scienceblogs.com/cognitivedaily/) was closed a short while after we started our research (January 2010). Hence, even though one of its authors continues to be active on Twitter, we classified Cognitive Daily as having no Twitter account. The blog Dinosaur Tracking (http://blogs.smithsonianmag.com/dinosaur/) had no Twitter account, but its author, Brian Switek, had an account for his other blog (also in our sample) Laelaps (http://www.wired.com/wiredscience/laelaps; @Laelaps) (See Figure 2).

**Figure 3:**
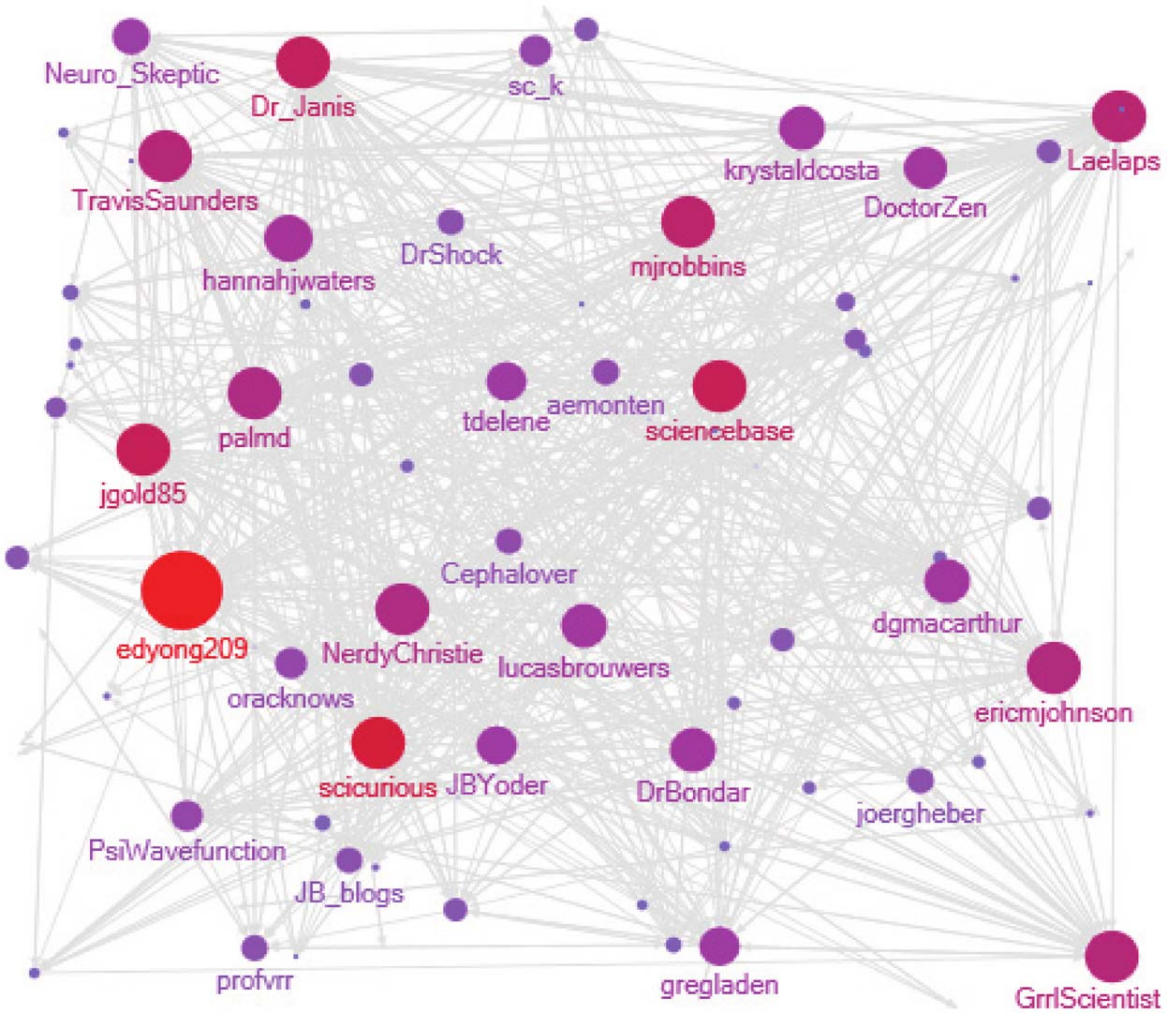
Twitter interconnections – followers.

We identified 101 Twitter accounts. The Twitter accounts were interconnected as can be seen in Figure 3. Only ten accounts did not follow any account from the sample, and only 18 accounts had no followers from the sample. The most followed account belonged to Ed Yong, who described himself as: “Science writer, creator of Not Exactly Rocket Science, freelance journalist” (@edyong209). He had 51 followers in our dataset, and he followed 24 of the bloggers in the sample. He had 11,638 followers and follows 778 Twitter accounts altogether. The maximum number of twitter accounts followed from among the sample was 39 by Peter Janiszewski (@Dr_Janis), co-founder of ScienceOfBlogging.com and ResearchBlogging.org editor. He followed 31 accounts in our sample. Altogether he followed 1,543 accounts and was being followed by 2,370 followers (as of October 2^nd^, 2011). In Figure 3 only the Twitter account names of users that were followed by 10 or more followers from our sample are displayed (38 accounts), the size and color of the nodes are proportional to the number of followers. The directed edge from node A to node B represents that A follows B. Thirty-eight accounts were being followed by ten or more bloggers from the sample. There were 28 accounts that both followed and were being followed by ten or more bloggers from our sample.

Note that blogs which are part of a network (e.g. Scientific American blogs) can spread their posts through the network’s Twitter account, which usually has a larger number of followers than an individual blogger (Science Blogs, @ScienceBlogs, have about 7,600 followers, Wired science blogs, @wiredsciblogs, have about 4,000 and Scientific American blogs, @sciamblogs, about 1,200). The RB Twitter account, @ResearchBlogs, (about 4,000 followers) automatically tweets every new post aggregated in RB (All network accounts were checked on October 2^nd^, 2011). The Technorati (Technorati.com) ranking showed that on October 2^th^, 2011 five of the blogs in our sample (Gene Expression, Not Exactly Rocket Science, Uncertain Principles, Pharma Strategy Blog and Greg Laden’s Blog) were ranked among the top 100 science blogs. All of these blogs have Twitter accounts. Gene Expression (@razibkhan) had 1,523 followers, Not Exactly Rocket Science (@edyong209) had 11,638, Pharma Strategy Blog (@MaverickNY) had 6,187, Uncertain Principles (@orzelc) had 830 and Greg Laden’s Blog (@gregladen) had 2,941 followers. While the numbers of followers vary widely, it seems all of the top bloggers in our sample also disseminate information via Twitter to a relatively large number of followers.

### Language

English is the dominant language of the science blogs in the study. Out of the 126 blogs in the sample 108 (86%) were written in English, 6 (5%) in Spanish, 5 (4%) in Portuguese, 4 (3%) in German, 2 (1%) in Polish and one (1%) in Chinese.

### Journals

The references appearing in the last five blog posts up to March 1^st^, 2011 in each of the 126 blogs were extracted. This resulted in 913 references to articles appearing in 429 journals, 9 references to articles uploaded to arxiv.org, 3 references to conference proceedings and 2 references to books. The distribution of the number of times journals were referenced appears in Table 3.

**Table 3:** Number of references per journal cited in the 5 most recent blog posts for the 126 blogs.

No. of references to journal	# journals	% journals
10 times or more	9	2
9 times	2	1
7 times	2	1
6 times	3	1
5 times	8	2
4 times	14	3
3 times	29	7
Twice	57	13
Once	305	70

### Subject Categories

For each of the journals that was referenced twice or more we identified the JCR subject category/categories they belong to (601 articles). Only 4 journals were not in ISI’s JCR for 2010. Based on the JCR journal categorization, the articles were classified into seven main classes (see Table 4). In a few cases the journal was categorized into more than one main category.

**Table 4:** Subject categories for journals cited at least twice in the 5 most recent blog posts for the 126 blogs with multidisciplinary papers manually classified into categories.

Main category	# papers	% papers
Life Sciences	286	43
Psychology, Psychiatry, Neurosciences,Behavioral Sciences	152	23
Medicine	113	17
Sciences	70	10
Social Science & Humanities	27	4
Computer Science & Engineering	18	3
Other	5	1

We manually classified multidisciplinary papers according to the same categories, based on their titles, abstracts and key words assigned to them by the journals (if any) and added their relative proportion to each category (Table 4). The distribution of the subject categories of the reviewed articles more or less coincides with the blog categorization, which is not surprising (see Table 1). Still, there are several differences, for example the percentage of social science papers that are reviewed (4%) is lower than the percentage of social science blogs (5%), and the percentage of life science papers (43%) is slightly higher than the percentage of life science blogs in the sample (39%).

### Most Blog-cited Journals

Science, Nature and PNAS are the highest-placed journals in the JCR multidisciplinary category and the most indexed in the online scientific reference manager Mendeley. These journals are the most “blog cited” in our sample as well (see Table 5). All the most cited journals in the sample were in the first quartile of their JCR category, thus there seems to be a clear trend toward reviewing papers appearing in high impact journals. This could be viewed as the rich-get-richer phenomenon; papers in high impact journals get more attention in the scientific blogosphere. The difference between publication volumes might also be an advantage for journals which publish more items. Another possibility is that RB bloggers read papers from lower impact journals as well, but review papers from higher impact journals because they consider these papers more “deserving” to be reviewed and exposed. Another hypothesis is that since mainstream media often report on papers from high-ranking, reputable journals [41]–[42] bloggers might focus on the same papers in order to offer their own analysis and interpretation. The findings further validate those of a previous study [26] which showed a preference for citing papers from top-ranking journals in RB posts about chemistry.

**Table 5:** Most cited journals according to number of blog citations, JCR category and JCR ranking in 2010.

Journal	JCR Category	JCR ranking in 2010	Number of blog citations
Science	Multidisciplinary Sciences	2/57	61
Nature	Multidisciplinary Sciences	1/57	53
Proceedings of the National Academy of Sciences	Multidisciplinary Sciences	3/57	43
PLoS One	Biology	12/85	37
Psychological science	Multidisciplinary Psychology	7/120	16
Proceedings of the Royal Society B- Biological Sciences	Biology, Ecology, Evolutionary Biology	9/85, 13/129, 9/45	11
BMJ- British Medical Journal	Medicine, General & Internal	6/151	10
JAMA-Journal of the American Medical Association	Medicine, General & Internal	3/151	10
Physical Review Letters	Multidisciplinary Physics	5/80	10
Biology Letters	Biology, Ecology, Evolutionary Biology	14/85, 28/129, 17/45	9
Journal of Neuroscience	Neurosciences	17/237	9
Cell	Biochemistry & Molecular Biology, Cell Biology	1/286, 2/177	7
NEJM- New England Journal of Medicine	Medicine, General & Internal	1/151	7
Journal of personality and social psychology	Social Psychology	3/56	6
Molecular biology and evolution	Biochemistry & Molecular Biology,Evolutionary Biology, Genetics & Heredity	49/286, 7/45, 20/156	6
Pediatrics	Pediatrics	1/107	6

### Education

In order to find out the bloggers’ level of education we searched for their personal information on the Web in the manner described in the methods section. In addition, we sent emails to those bloggers we had not been able to extract their education level from information publicly available on the Web. Seven bloggers did not have an email address, and therefore we were only able to send email to sixteen of our unknown bloggers, and received seven answers. Some of the bloggers might have wanted to preserve their anonymity and therefore did not reply to our emails.

**Figure 4:**
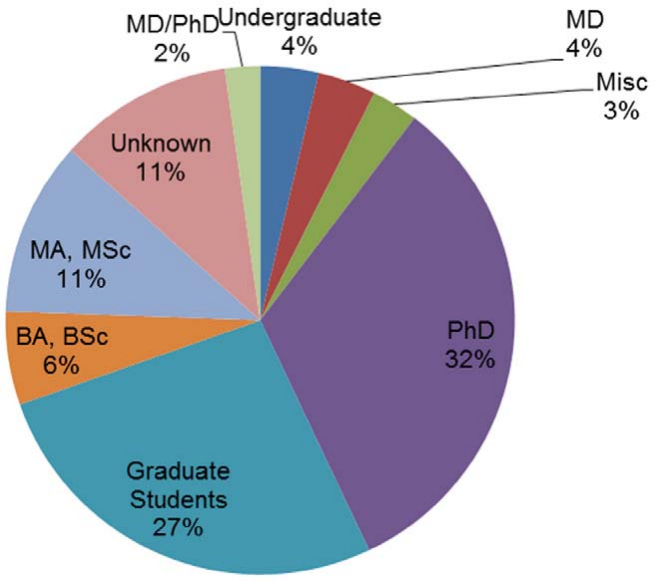
Distribution of bloggers’ education levels.

The science bloggers in our sample were highly educated. Five bloggers (4%) were undergraduates, another 5 (4%) were Medical Doctors (MD), 8 (6%) had a BA or a BSc, 15 (11%) had an MA or an MSc, 36 (27%) were graduate students, 3 (2%) had both a medical degree and a PhD., (MD/PhD.) 44 had a Ph.D. (32%), 4 (3%) had other degrees and 15 (11%) remained unknown (Figure 4).

**Figure 5:**
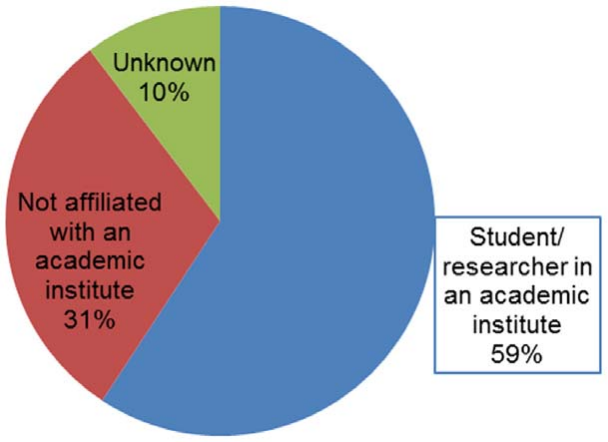
Affiliation with an academic institute.

As Figure 5 shows, most of the bloggers (59%) were either students or researchers in an academic institute. Less than a third (30%) were not affiliated with an academic institute, and 10% remained unknown. It is possible that the bloggers, due to their involvement in the academy, see the citation as a valuable mechanism even when writing in social media.

### Anonymity

Bloggers who do not supply a name or only supply a nickname or first name were referred to as anonymous. It must be noted that we have not made inquiries about the authenticity of names; therefore, it is possible that names which appear to be authentic were pseudonyms. If bloggers linked to another page under their full name (such as an article they wrote or their Twitter account) we considered the blogger to be non-anonymous. Most bloggers chose to blog under their full name. Out of the 135 bloggers in our sample only 22 (16%) blogged anonymously.

### Limitations

Our study has several limitations: blogs are dynamic by nature. They open, close, join a network or leave it, add authors or lose them at a rapid pace. Hence our blogs may have changed since they were assessed. Moreover, we assumed the bloggers’ profiles to be authentic and up-to-date, but could not fully verify this. We focused on non-commercial blogs from one aggregator with 1–2 authors, and chose fairly established blogs. Our sample included only blogs which cite their sources in an academic style and post to the RB aggregator. Our characterization might therefore only be true for the blogs in our sample, rather than the general science blogs population. In particular, our sample may have biases towards disciplines in which RB is well known and towards bloggers that promote their blogs by submitting them to RB.

### Summary and Conclusions

Our aim was to characterize blogs and bloggers who write about academic, peer-reviewed research. Given the familiarity of the bloggers in our sample with bibliographic citations, it is no wonder that over sixty-five percent of them are graduate students, PhDs, MDs or MD/PhDs and that 59% are currently affiliated with an academic institute. The bloggers regularly cite well-known, high-impact journals which publish multidisciplinary science (Science, Nature and PNAS) and leading niche journals, (e.g. New England Journal of Medicine, Journal of Neuroscience). This confirms and adds to Groth and Gurney’s findings [26] that RB posts about chemistry often cite papers from high-impact journals either because of these papers’ scientific importance or because of the reputation of the journals. In addition, the bloggers might be reflecting the mainstream media’s tendency to cover papers from leading journals in order to criticize media coverage of scientific issues. In a post called “Dear Newspapers: Individual Studies Do Not Exist In A Vacuum” the blog Obesity Panacea (http://blogs.plos.org/obesitypanacea/) cited papers from PLoS One and BMC Public Health with contradicting conclusions, in order to make the claim that the media’s tendency to report a single study at a time can cause public confusion [43]. Life science is the most popular blog category (39%) as well as the biggest subject category (43%), much like in the current RB post population (about 9,000 of 20,600, or around 43% of the posts, were tagged under “biology” in September 2011), confirming a previous analysis showing that the “biology” tag comprised 32% of the RB tags [28]. This high number of life science blogs and posts may be connected to the high number of post-doctorate positions in life science and medicine [29], as well as to the rising number of life science doctorates awarded [31]. Authors and readers from other disciplines may also not be as familiar with RB as those from the life sciences.

Most (84%) bloggers apparently blog under their real name. This high percentage suggests that science bloggers see their blog, if not as a career enhancer, then at least as career-neutral. RB aggregates blogs in several languages, but the bloggers mostly (86%) blog in English. Seventy-two percent of the blogs have active Twitter accounts. In comparison, only 2.5% of the academics studied by Priem and colleagues [44] had active Twitter accounts. The high percentage of Twitter accounts belonging to blogs and the number of accounts following popular blogs show that many of the bloggers are information disseminators in more than one social medium. Twenty-eight Twitter accounts belonging to bloggers in our sample both follow and are being followed by ten or more bloggers from the sample, showing that there is a core of quite well connected bloggers. Moreover, since 90% of accounts followed another account from the sample, and 82% of accounts had a follower from the sample, it seems reasonable to view the Twitter accounts as at least loosely interconnected.

We found a lack of gender balance in the science blogging gender distribution, with 72% of the blogs being written by one or two male authors. This is in line with studies of Wikipedia [32]–[33] and about the general distribution of RB bloggers [34]. While RB is open to any kind of blogging which refers to peer-reviewed research, its highest tagging coverage is mostly in the science & engineering fields, in which women made up in 2006 only about 40% of the PhDs and 29% of the full-time doctoral faculty [31], [45]. Fields such as education, history and literature are only represented as subtags.

In conclusion, the sample’s science blogs share characteristics with other means of scientific discourse. We believe that tracking and recording this communication will become a part of future research evaluation metrics.

## References

[1] Priem J, Taraborelli D, Groth P, Neylon C (2010). Alt-metrics: a manifesto.. http://altmetrics.org/manifesto/.

[2] Blackwell PK, Kochtanek TR (1981). An iterative technique for document-retrieval using descriptors and relations.. Proceedings of the American Society for Information Science.

[3] Merton RK (1973). The normative structure of science..

[4] Cronin B (1984). The citation process..

[5] Garfield E (1979). Citation indexing: its theory and application in science, Technology, and Humanities..

[6] Wouters P (1999). The citation culture..

[7] Gargouri Y, Hajjem C, Larivière V, Gingras Y, Carr L (2010). Self-Selected or Mandated, Open Access Increases Citation Impact for Higher Quality Research.. PLoS ONE.

[8] Vaughan L, Shaw D (2003). Bibliographic and Web citations: What is the difference?. Journal of the American Society for Information Science and Technology.

[9] Vaughan L, Shaw D (2005). Web citation data for impact assessment: A comparison of four science disciplines.. Journal of the American Society for Information Science and Technology.

[10] Kousha K, Thelwall M (2007). How is science cited on the web? A classification of Google unique web citations.. Journal of the American society for Information Science and Technology.

[11] Ashlin A, Ladle RJ (2006). Environmental science adrift in the blogosphere..

[12] Batts SA, Anthis NJ, Smith TC (2008). Advancing Science through Conversations: Bridging the Gap between Blogs and the Academy.. PLoS Biol.

[13] Amsen E (2006). Who benefits from science blogging?. Hypothesis.

[14] Kjellberg S (2010). I am a blogging researcher: Motivations for blogging in a scholarly context.. First Monday.

[15] Walker J, InBruns A, Jacobs J (2006). Blogging from inside the ivory tower..

[16] Goldstein A (2009). Blogging evolution..

[17] Kouper I (2010). Science blogs and public engagement with science: Practices, challenges and opportunities. Journal of Science Communication 9:1 A02.. http://jcom.sissa.it/archive/09/01/Jcom0901(2010)A02/Jcom0901(2010)A02.pdf.

[18] Kovic I, Lulic I, Brumini G (2008). Examining the medical blogosphere: an online survey of medical bloggers. J Med Internet Res..

[19] Marcus A, Oransky I (2011). Science publishing: The paper is not sacred. Nature..

[20] Wolfe-Simon F, Switzer Blum J, Kulp TR, Gordon GW, Hoeft SE (2010). A bacterium that can grow by using arsenic instead of phosphorus. Science [online early access].. http://www.sciencemag.org/content/332/6034/1163.short.

[21] Zivkovic B (2011). #Arseniclife link collection. Blog Around the Clock.. http://blogs.scientificamerican.com/a-blog-around-the-clock/2011/09/30/arseniclife-link-collection/.

[22] Jha A, Kingsland J (2010). Fallout from Nasa’s ‘arsenic bacteria’. The Guardian.. http://www.guardian.co.uk/science/2010/dec/02/nasa-life-form-bacteria-arsenic.

[23] CBC News (2010). NASA’s arsenic microbe science slammed. CBC News.. http://www.cbc.ca/news/technology/story/2010/12/06/arsenic-microbe-dna-nasa-wolfe-simon.html.

[24] Munger, D (2010). Death for “Arsenic-Based Life”? Seed Magazine.. http://seedmagazine.com/content/article/death_for_arsenic-based_life/.

[25] Redfield, RJ (2011). Comment on “A Bacterium That Can Grow by Using Arsenic Instead of Phosphorus”. Science.. http://www.sciencemag.org/content/332/6034/1149.8.full.

[26] Groth P, Gurney T (2010). Studying scientific discourse on the Web using bibliometrics: A chemistry blogging case study..

[27] Smith M, Milic-Frayling N, Shneiderman B, Mendes Rodrigues E, Leskovec J (2010). http://nodexl.codeplex.com/from.

[28] Munger D (2010). Rethinking the topic tags on Research Blogging.. http://researchblogging.org/news/?p=1864.

[29] National Science Foundation, Division of Science Resources Statistics (a) (2010). Doctorate recipients from U.S. universities: 2009, special report NSF 11–306. Arlington, VA.. http://www.nsf.gov/statistics/nsf11306/nsf11306.pdf.

[30] Bonetta L (2007). Scientists enter the blogosphere.. Cell.

[31] National Science Foundation, Division of Science Resources Statistics (b) (2010). Science & engineering indicators: 2010. Chapter 2. Higher Education in Science and Engineering.. http://www.nsf.gov/statistics/seind10/c2/c2s4.htm.

[32] Glott R, Schmidt P, Ghosh R, report Technical Wikipedia survey – overview of results.. UNU- MERIT.

[33] Lam SK, Uduwage A, Dong Z, Sen S, Musicant DR (2011). WP:Clubhouse?.

[34] Munger D (2010). Blogging out of balance. Seed Magazine.. http://seedmagazine.com/content/article/blogging_out_of_balance/.

[35] Sneyd E (2010). The Guardian launches science blogs network.. http://www.themediabriefing.com/article/2010-08-31/the-guardian-launches-science-blogs-network.

[36] Allen L (2010). Announcing PLoS Blogs.. http://blogs.plos.org/plos/2010/09/announcing-plos-blogs/.

[37] Mason B (2010). Meet the New Wired Science All-Star Bloggers.. http://www.wired.com/wiredscience/2010/09/new-wired-science-blogs-network/.

[38] Scheer R (2011). Blog Network Launches on ScientificAmerican.com.. http://www.scientificamerican.com/pressroom/pr/release.cfm?site=sciam&date=2011-07-05.

[39] Twitter (2011). One hundred million voices.. http://blog.twitter.com/2011/09/one-hundred-million-voices.html.

[40] Priem J, Costello, K (2010). How and why scholars cite on Twitter..

[41] Conrad P (1999). Uses of expertise: sources, quotes, and voice in the reporting of genetics in the news.. Public Understing of Science.

[42] Shema H, Bar-Ilan J (2010). Sources of health articles in Israeli news sites.. Meida’at.

[43] Saunders T (2011). Dear newspapers: individual studies do not exist in a vacuum. Obesity Panacea.. http://blogs.plos.org/obesitypanacea/2011/12/08/dear-newspapers-individual-studies-do-not-exist-in-a-vacuum/.

[44] Priem J, Costello K, Dzuba T (2011). First-year graduate students just wasting time? Prevalence and use of Twitter among scholars. Presented at the Metrics 2011 Symposium on Informetric and Scientometric Research, New Orleans, LA, USA.. http://jasonpriem.org/self-archived/5uni-poster.png.

[45] Science Foundation, Division of Science Resources Statistics (b) (2010). Science & engineering indicators: 2010. Chapter 5. Higher Education in Science and Engineering.. http://www.nsf.gov/statistics/seind10/c5/c5h.htm.

